# Synchronization of megathrust earthquakes to periodic slow slip events in a single-degree-of-freedom spring-slider model

**DOI:** 10.1038/s41598-019-44684-4

**Published:** 2019-06-04

**Authors:** Makiko Ohtani, Nobuki Kame, Masao Nakatani

**Affiliations:** 1 Geological Survey of Japan, AIST, Central 7, 1-1-1 Higashi, Tsukuba, Ibaraki 305-8567 Japan; 2 Earthquake Research Institute, The University of Tokyo, 1-1-1 Yayoi, Bunkyo-ku, Tokyo 113-0032 Japan

**Keywords:** Natural hazards, Seismology

## Abstract

Recently recognized slow slip events (SSEs) recurring in the deeper extensions of seismogenic zones along plate boundaries are drawing attention to their potential for triggering megathrust earthquakes that rupture the entire seismogenic zone. We describe how earthquakes simulated in a single-degree-of-freedom model are synchronized to the rhythm of imposed periodic SSEs. The time lag *t*_*Q*_ from the one most recent SSE to a seismic event varies with system parameters and may take a broad range of eligible values between 0 and *T*_*SSE*_ (SSE recurrence period). Earthquakes were found to synchronize with SSEs in various patterns depending on the proportion of SSE-driven loading within an SSE cycle, the recurrence period of the SSEs, and the duration of the SSEs, although synchronization itself remained a prevalent feature. Asynchrony was found only for long SSE durations.

## Introduction

Global Navigation Satellite System (GNSS) networks, being deployed recently, have revealed the presence of slow slip events (SSEs) in the deeper extensions of seismogenic zones^1–4^. SSEs often occur in a brittle-to-ductile transition zone just below the brittle zone where large earthquakes occur, so they are believed to be affecting earthquake occurrences to some extent or other^5^. It is therefore essential to study possible effects of SSEs on earthquake timings.

The SSEs are characterized by their recurrent nature. It is known, for example, that SSEs measuring about 20 cm in slip amounts occurred three times over 30 years at an identical location beneath Lake Hamana along the Nankai Trough (Tokai SSEs)^6,7^. SSEs with slip amounts of 20–30 cm are also occurring, more steadily, every 6–7 years beneath the Bungo Channel west of Shikoku (Bungo SSEs)^8,9^. SSEs measuring some 2 cm in slip amounts are recurring with a period of 14 months or so in Cascadia^2^.

A deep, repeating SSE of a similar type is known to have actually triggered an earthquake. In the Guerrero region of Mexico, where SSEs recur every 3–4 years, an earthquake occurred in an area adjacent to the location of an ongoing SSE in 2014 (Papanoa earthquake). It is believed the seismic event was triggered by the SSE^10^.

A variety of models have so far been used to study how an earthquake fault responds to stress perturbations from nearby events^11–13^. Those models used the steady increase of fault stress to represent tectonic loading, and a stress perturbation was imposed at an arbitrary magnitude and timing. These studies consider only one-time stress perturbation event, so the impact of the history of SSEs recurring throughout a seismic cycle cannot be evaluated. The present study uses a single-degree-of-freedom spring-slider model to represent the impact of repeating SSEs on an earthquake fault as changes in the pulling velocity of the spring (episodic-loading model) and to study their impact on the timing of earthquakes.

In our numerical experiments, we assume different values of the characteristic slip distance *L*, which is a key fault-surface friction parameter, and focus on the recurrence intervals of earthquakes and their timing (phase difference) with respect to SSE recurrence. The fault toughness is proportional to *L*, and the earthquake recurrence interval grows continuously in proportion to *L* under tectonic, steady loading^14^. The recurrence interval in that situation (steady-loading model) shall be denoted by *T*_0_, the natural characteristic period. In the episodic-loading model, by contrast, we have found that earthquakes are synchronized with SSEs, with their recurrence interval *T* behaving characteristically of a synchronization phenomenon. Surprisingly, synchronization occurred even by the repetition of very small SSEs, and we could not reach a physiological understanding such as a characteristic evolution of the stress and the strength leading to synchronization. However, through experiments with various patterns of SSEs, we could find that the overall behavior of this synchronization phenomenon makes good sense in a lot of ways.

An earlier study pointed out that synchronization occurs in a spring-slider model^15^. It was numerically shown how more than one block engages in stick slip with one and the same period in a system of weakly interacting blocks that are mutually coupled via springs and have much the same characteristic periods when they are free from the influence of the other blocks. Another study attributed the observed clustering in time of great earthquakes in a given region to a synchronization phenomenon^16^. The situation assumed in the model study corresponds to that case. Unlike these studies, however, our study deals with earthquakes that are affected by a phenomenon (SSEs) with a much shorter period than the earthquakes themselves. We demonstrate that synchronization still occurs in that case.

## Results

### Model

**Figure 1:**
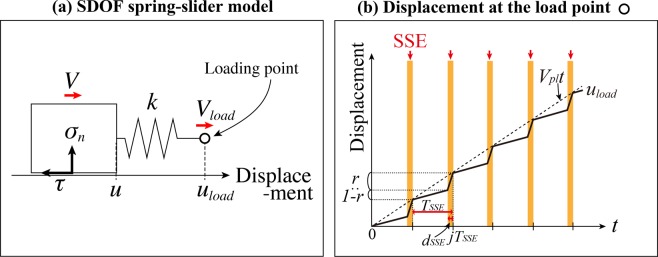
(**a**) A schematic diagram showing a single-degree-of-freedom spring-slider model. (**b**) Evolution of the displacement *u*_*load*_(*t*) of the spring’s load point (open circle in **a**) with respect to time *t*. Orange stripes indicate ongoing SSEs (from *t* = *jT*_*SSE*_ − *d*_*SSE*_ to *t* = *jT*_*SSE*_, where *j* is an integer).

To simulate plate-boundary megathrust earthquakes, we assume a system of a block being pulled on a frictional floor via a spring at velocity *V*_*load*_ (Fig. 1a). Recurrent SSEs in the deeper extension of an earthquake fault are modeled as changes in the load-point velocity (Fig. 1b; pulling displacement *u*_*load*_; *V*_*load*_ = *du*_*load*_/*dt*) so the impact of the repeating SSEs is integrated into the loading history. We conduct a quantitative study of dependence of the system behavior on the proportion *r* of the loading driven by SSEs within an SSE cycle, their recurrence period *T*_*SSE*_ and their duration *d*_*SSE*_. An SSE begins at time *t* = *jT*_*SSE*_ − *d*_*SSE*_ and ends at *t* = *jT*_*SSE*_ (*j*: integer). The *V*_*load*_ is set at *rV*_*pl*_*T*_*SSE*_/*d*_*SSE*_ when an SSE is going on and at (1 − *r*)*V*_*pl*_*T*_*SSE*_/(*T*_*SSE*_ − *d*_*SSE*_) during the rest of the time so the long-term pulling velocity remains at *V*_*pl*_, where *V*_*pl*_ = 5 cm/yr and *r* = 0–1. A step displacement *Δu*_*SSE*_ = *rV*_*pl*_*T*_*SSE*_ is imposed, however, when *d*_*SSE*_ = 0. The time evolution of the slip is calculated according to the Method.

### Synchronization of earthquakes to repeating SSEs

**Figure 2:**
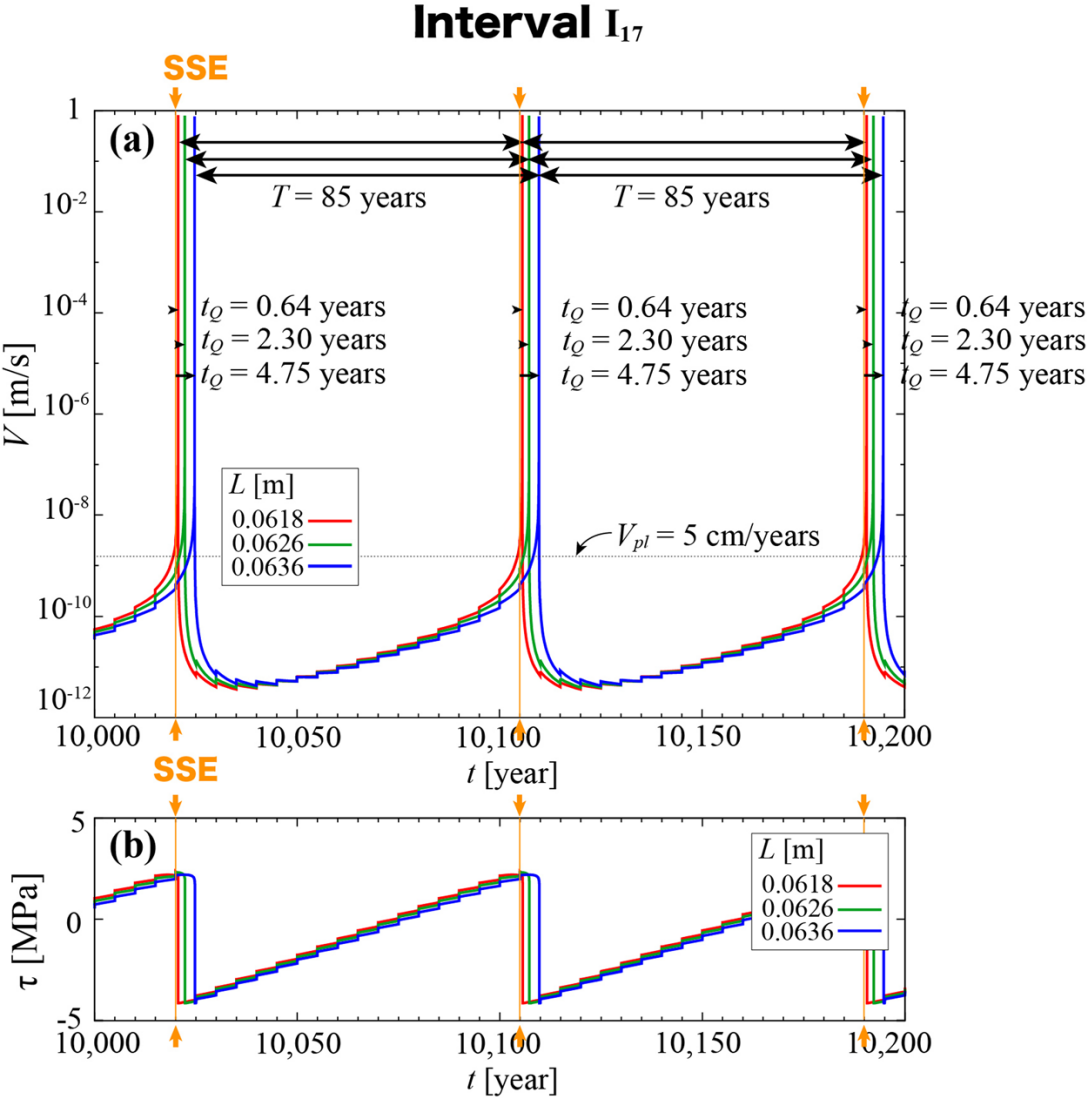
Time evolutions of (**a**) *V* [m/s] and (**b**) *τ* [MPa] for *r* = 0.5, *T*_*SSE*_ = 5 yr and *d*_*SSE*_ = 0, with red, green and blue showing the results for *L* = 0.0618 m, 0.0626 m and 0.0636 m, respectively, all within interval I_17_. In all cases, *T* = 85 yr. The time lag *t*_*Q*_ [yr] from the onset of the most recent SSE (orange; *t* = 10,020 yr, 10,105 yr and 10,190 yr) to the seismic event is also indicated for each case in the figure.

In this subsection, we illustrate the example of a numerical test with *r* = 0.5, *T*_*SSE*_ = 5 yr and *d*_*SSE*_ = 0 to outline the synchronization phenomenon observed. The red curves in Fig. 2a,b show the time evolution of the slip rate *V* and stress *τ*, respectively, of the block in the case of *L* = 0.0618 m. The step-like growths in *V* and *τ* during the interseismic period are attributable to SSEs that occur every 5 years. In this case, earthquakes were found always to recur at an interval of *T* = 85 yr. A steady-loading model (*V*_*load*_ = *V*_*pl*_) with the same *L* value gives, by contrast, *T*_0_ = 83.87 yr. The episodic-loading model gave a longer earthquake recurrence interval for an identical long-term loading rate.

Figure 2 also shows, in green and blue, the results for larger *L* values of 0.0626 m and 0.0636 m, respectively. In both cases, *T* remained unchanged at 85 yr. As we have stated above, *T*_0_ grows continuously in proportion to *L* in the steady-loading model. When *L* = 0.0636 m, *T*_0_ = 86.31 yr, which means the recurrence interval in the episodic-loading model remains fixed and is now, contrary to the case of *L* = 0.0618 m, smaller than *T*_0_. And interestingly, the persistent value of *T* = 85 yr is exactly 17 times *T*_*SSE*_, which means the earthquakes are synchronized to the periodicity of the SSEs. This suggests a so-called entrainment phenomenon is at work. The fact that earthquakes see the rhythm of SSEs means that the timings of individual earthquakes is affected not only by the most recent SSE but also by the earlier sequence of SSEs.

**Figure 3:**
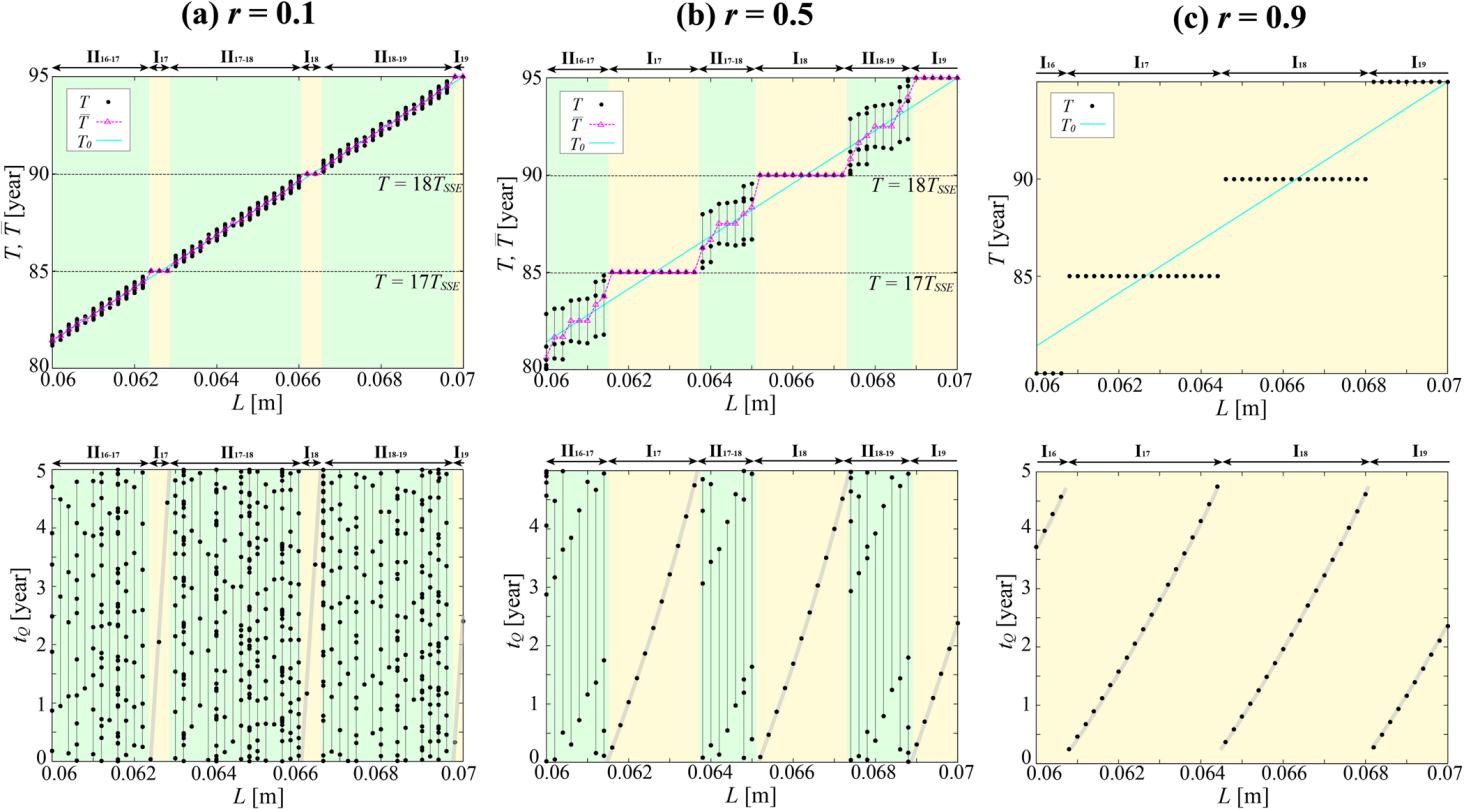
The *T*, $\bar{T}$ and *t*_*Q*_ [yr] in the case of *T*_*SSE*_ = 5 yr and *d*_*SSE*_ = 0 under (**a**) *r* = 0.1, (**b**) *r* = 0.5, (**c**) *r* = 0.9. In the top panels, circles and triangles denote *T* and $\bar{T}$, respectively, whereas the bottom panels show *t*_*Q*_. The *T* alone is shown for *r* = 0.9, because *T* = $\bar{T}$ in this case. The light-blue lines in the top panels show the corresponding *T*_0_ in the steady-loading model. In each panel, the light-yellow and light-green stripes indicate regime (i) (intervals I_*m*_) and regime (ii) (intervals II_*m*−(*m*+1)_), respectively.

Figure 3b top shows the response of *T* over a broader range of *L*. Plotted in the panel are all the 200 or so values of *T* that emerged during the time interval *t* = 10,000 yr–30,000 yr in each of the simulation runs with different *L* values, which were sampled at intervals of 2 × 10^−4^ m. The light-blue lines in the figure show, for reference, the values of *T*_0_(*L*) in the steady-loading model. The results came in two patterns: regime (i), where a unique *T* value is determined for a given *L*, and regime (ii), where more than one *T* value is ascribable to a given *L*.

The interval *L* = 0.0616 m–0.0636 m, for example, falls in regime (i). This interval is centered on a value of *L*, which we shall call *L*_17_, at which the natural characteristic period *T*_0_ is exactly 17 times the SSE recurrence period (*T*_0_(*L*_17_) = 17*T*_*SSE*_). In this interval, which we shall call I_17_, *T* remains fixed at 85 yr = 17*T*_*SSE*_, and the periodicity of earthquakes is synchronized to the periodicity of SSEs. The cases shown in Fig. 2 all fall in the same interval I_17_.

A range of greater *L* values, at *L* = 0.0638 m–0.0650 m, falls in regime (ii). More than one value of *T*, lying between 17*T*_*SSE*_ and 18*T*_*SSE*_, emerges at each *L* during a single simulation run (we shall call this interval II_17−18_). When *L* = 0.0644 m, for example, there are two alternating varieties of *T*, namely *T*_1_ = 86.43 yr and *T*_2_ = 88.57 yr, which add up to 35*T*_*SSE*_. In other words, an earthquake occurrence pattern is repeating with a period of *P* = *T*_1_ + *T*_2_ = 35*T*_*SSE*_, so earthquakes can be deemed synchronized to SSEs. The number of earthquakes per repeat cycle shall be called as *n*_*EQ*_. In this case, *n*_*EQ*_ = 2; period-doubling bifurcation^17^ occurred. When *L* = 0.0650 m, *n*_*EQ*_ = 3 varieties of the recurrence interval, namely *T*_1_ = 88.76 yr, *T*_2_ = 89.55 yr and *T*_3_ = 86.69 yr, repeat periodically. An earthquake occurrence pattern is repeating with a period of *P* = *T*_1_ + *T*_2_ + *T*_3_ = 53*T*_*SSE*_. A relationship *P* = *n*_*SSE*_*T*_*SSE*_ (*n*_*SSE*_: the number of SSEs per repeat cycle) always holds under regime (ii), so earthquakes can be deemed synchronized to SSEs under regime (ii), just as they are under regime (i). With *L* growing further, regime (i) sets in again at *L* = 0.0652 m–0.0672 m, with *T* fixed at 18*T*_*SSE*_ (we shall call this interval I_18_).

This illustrates how regimes (i) and (ii) alternate with each other with changing system parameter *L*. With growing *L*, in other words, *T* continues to cling around *T*_0_(*L*) as it also grows, and goes alternately through an interval I_*m*_ with *T* = *mT*_*SSE*_ and an interval II_*m−*(*m*+1)_ with *mT*_*SSE*_ < *T*_*k*_ (1 ≦ *k* ≦ *n*_*EQ*_) < (*m* + 1)*T*_*SSE*_, where *m* is an appropriate integer.

In both regimes (i) and (ii), *P* = *n*_*EQ*_ $\bar{T}$ = *n*_*SSE*_*T*_*SSE*_, where $\bar{T}$ is the mean earthquake recurrence interval, so *m* = *n*_*SSE*_/*n*_*EQ*_, *n*_*EQ*_ = 1 under regime (i) and *n*_*EQ*_ > 1 under regime (ii). Regime (ii) is so-called high order synchronization (HOS) or *n:m* synchronization^18^. The triangles in Fig. 3b top show values of $\bar{T}$, which grows monotonically, and in steps, with growing *L*. Apart from what looks like stair treads under regime (i)—a feature that is also seen in the variation pattern for *T*—the panel also shows the presence of fine, step-like features in $\bar{T}$ within regime (ii). This type of behavior is characteristic of synchronization phenomena and is known by the name of the devil’s staircase^19^.

Let us introduce *ΔT*_*max*_, or the upper limit of |*T*_*k*_ − *T*_0_| (amount of the deviation of an earthquake recurrence interval from the natural period *T*_0_) within the entire experimented range *L* = 0.06–0.07 m as an indicator for measuring how far the values of *T*, realized under synchronization effects, can deviate from *T*_0_. The |*T*_*k*_ − *T*_0_| was found to peak at both ends of each interval I_*m*_, and *ΔT*_*max*_ = 1.51 yr = 0.30*T*_*SSE*_ in Fig. 3b. The *ΔT*_*max*_ can be interpreted as representing the magnitude of the “entrainment potential” of SSEs for entraining earthquake recurrence intervals into periodicity of a different nature.

The variation pattern for *T* may be interpreted as follows if we assume that SSEs cannot alter the periodicity of earthquakes above and beyond their entrainment potential. The *T* can only vary within the range *T*_0_ − *ΔT*_*max*_ < *T* < *T*_0_ + *ΔT*_*max*_, and if any *mT*_*SSE*_ values are available within that range, *T* is entrained into the closest of those *mT*_*SSE*_ values, and regime (i) sets in. When no *mT*_*SSE*_ value is available within that range, by contrast, the occurrence intervals keep varying within that eligible range and form a sequence of more than one seismic event, whose intervals sum up to an integer multiple of *T*_*SSE*_. That sequence serves as a repeat unit for achieving synchronization to SSEs. This is regime (ii). Regime (i) amounts, so to speak, to direct synchronization to the periodicity of SSEs, whereas regime (ii), with a weaker degree of synchronization than in (i), could be described as roundabout (indirect) synchronization. We here define a phenomenological parameter *S*, the proportion of the *L* intervals that realize regime (i), which is a measure of the prevalence of direct synchronization. In this case, *S* = 0.60.

Under the mechanism of entrainment proposed above, interval I_*m*_ should be [*L*_*m*_ − *ΔL*, *L*_*m*_ + *ΔL*], where *T*_0_(*L*_*m*_) = *mT*_*SSE*_, and ${T}_{0}({L}_{m}\pm {\rm{\Delta }}L)={T}_{0}({L}_{m})\pm {\rm{\Delta }}{T}_{max}$. Therefore, it is expected that

1 $$S=2{\Delta }{T}_{max}/{T}_{SSE}.$$

Interval I_18_, for example, covers the range *L* = 0.0652 m–0.0672 m. The widths of similar *L* ranges remained largely invariant regardless of the index *m*, and *S* = 0.60. That, combined with *ΔT*_*max*_ = 1.51 yr = 0.30*T*_*SSE*_, confirms that Eq. (1) is met.

Let us next study the time lag *t*_*Q*_ from the onset of the most recent SSE to a seismic event. In the case of Fig. 2 (interval I_17_), *t*_*Q*_ = 0.64 yr, 2.30 yr and 4.75 yr when *L* = 0.0618 m, 0.0626 m and 0.0636 m, respectively, with *t*_*Q*_ growing with increasing *L*. Each of these timing relations between the SSEs and earthquakes remained invariant for all seismic events that recurred within a single simulation run. The earthquakes ended up phase-locked to the SSEs at the same *T* and *t*_*Q*_ values even when the SSEs were shifted in occurrence time as part of the initial conditions.

Figure 3b bottom shows *t*_*Q*_ values over a broader range of *L*. As seen earlier in Fig. 2, *t*_*Q*_ grows with increasing *L* within an interval I_*m*_ along a slightly convex-downward curve shown in bold in the figure, and takes a broad variety of values ranging from 0 all the way up to *T*_*SSE*_. Within an interval II_*m−*(*m*+1)_, more than one *T* value corresponds to a given *L*, with each *T*_*k*_ associated with its own, different *t*_*Q*_ value. In both regimes (i) and (ii), the time lag *t*_*Q*_ was found to take a broad range of eligible values between 0 and *T*_*SSE*_. The earthquakes are synchronized to the SSEs, without, however, showing any strong tendency to occur shortly following an SSE.

Dependence of the system behavior on characteristics of the SSEs (their impact size *r*, recurrence period *T*_*SSE*_ and duration *d*_*SSE*_) will be studied in the following subsections.

### Effect of *r*

This subsection addresses dependence of the system behavior on the proportion *r* of the stress attributable to SSEs to all stress loaded on the fault during an SSE cycle. Apart from the above-described case of *r* = 0.5, we also studied the cases of *r* = 0.1 and 0.9, and the results are shown in Fig. 3a,c, respectively. The study found that earthquakes are synchronized with SSEs when *r* = 0.1 and 0.9 as well, with *T* and $\bar{T}$ generally growing with increasing *L* in following the “devil’s staircase” pattern, except the case of *r* = 0.9, when regime (ii) does not exist. Note regime (ii) under *r* = 0.1 (Fig. 3a top) does exhibit small steps, though not seen with the coarse sampling of *L* in this figure (see Supplementary for finer sampling).

When *r* = 0.1, 0.5 and 0.9, *S* = 0.12, 0.60 and 1.00, respectively. All *L* values are under regime (i) when *r* = 0.9, and regime (ii) emerges in ever increasing proportions as *r* becomes smaller. Also, *ΔT*_*max*_ = 0.32 yr (0.06*T*_*SSE*_), 1.51 yr (0.30*T*_*SSE*_) and 2.49 yr (0.50*T*_*SSE*_), respectively, which satisfies Eq. (1). Both *ΔT*_*max*_ and *S* grow in proportion to *r* until they flatten off at the upper limits of *S* = 1.00 and *ΔT*_*max*_ = 0.50*T*_*SSE*_, respectively, at *r* = 0.9.

All recurrence intervals are close to *T*_0_ when *r* is small. That is understandable, because a small *r* means only a small portion of the loads are driven by SSEs, so *ΔT*_*max*_, or the entrainment potential of the SSEs, is also small, and *T*, therefore, cannot deviate too far from the natural period *T*_0_.

It was also found that more *T* and *t*_*Q*_ values correspond to a single *L* under regime (ii) when *r* = 0.1 than when *r* = 0.5, so *n*_*EQ*_ and *n*_*SSE*_ can sometimes take very large values. Among the largest, for example, are *n*_*EQ*_ = 43 and *n*_*SSE*_ = 719 for *L* = 0.0616 m. Phase-locking, however, still persisted in that case, and earthquakes were still found to be synchronized with SSEs. It appears likely that large *n*_*EQ*_ values are required to form a repeat pattern under the constraint that, as stated above, *T* cannot deviate very far from *T*_0_ when *r* is small.

When *r* = 0.1 and 0.9, *t*_*Q*_ (Fig. 3 bottom) was found to grow with increasing *L* within an interval I_*m*_ along a slightly convex-downward curve, just like in the case of *r* = 0.5. In both regimes (i) and (ii), the *t*_*Q*_ values are distributed broadly across the range 0–*T*_*SSE*_, and the earthquakes exhibit no strong tendency to occur shortly following an SSE, even when SSE is large (*r* = 0.9).

### Effect of *T*_*SSE*_

**Figure 4:**
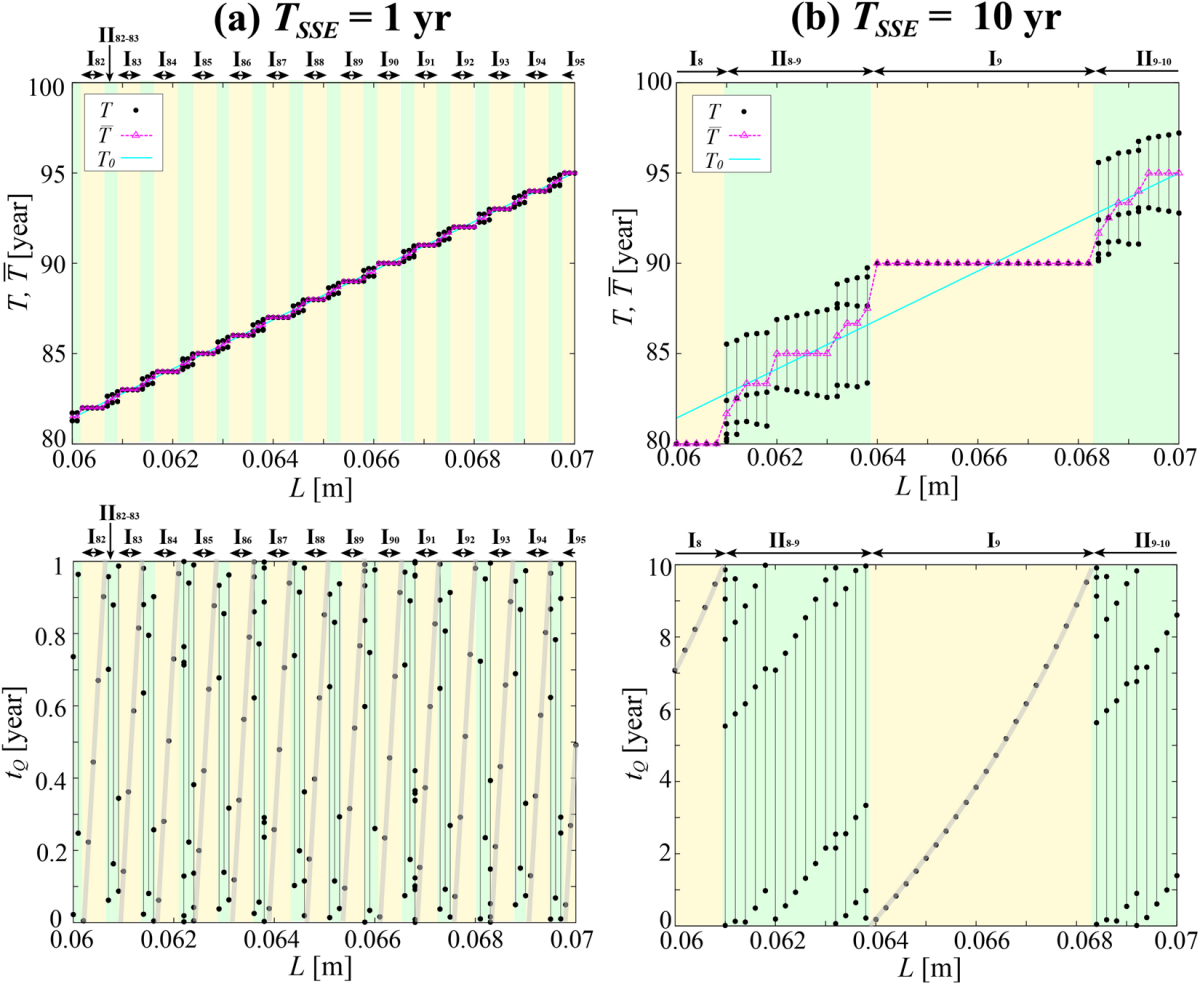
The *T*, $\bar{T}$ and *t*_*Q*_ [yr] in the case of *r* = 0.5 and *d*_*SSE*_ = 0 under (**a**) *T*_*SSE*_ = 1 yr and (**b**) *T*_*SSE*_ = 10 yr. See caption to Fig. 3 for details.

Let us next study how earthquake recurrence patterns vary with changes in the SSE recurrence period *T*_*SSE*_. We fixed *r* at 0.5 and *d*_*SSE*_ at 0 as we changed *T*_*SSE*_ from the 5 yr as before (Fig. 3b) to 1 yr and 10 yr. The results are shown in Fig. 4. Just like before, each *T*_*k*_ was found to be associated with a fixed, unique value of *t*_*Q*_, and phase-locking was confirmed for all *T*_*SSE*_ values. The *T* and $\bar{T}$ grow with increasing *L* as they go alternately through regimes (i) and (ii), and, just like in the earlier cases, the *t*_*Q*_ values were found to be distributed broadly across the eligible range of 0–*T*_*SSE*_. It was found that *S* = 0.59, 0.60 and 0.59, and *ΔT*_*max*_ = 0.30 yr, 1.51 yr and 3.14 yr for *T*_*SSE*_ = 1 yr, 5 yr and 10 yr, respectively. In other words, *ΔT*_*max*_ = 0.30*T*_*SSE*_, 0.30*T*_*SSE*_ and 0.31*T*_*SSE*_, respectively, which confirms Eq. (1).

The ratio *ΔT*_*max*_*/T*_*SSE*_ remains almost unchanged, and that is also true in the cases of other *r* values, which we are not showing in our figures here. This, combined with *ΔT*_*max*_ ∝ *r* found in the previous subsection, shows that *ΔT*_*max*_ ∝ *rT*_*SSE*_. This means the entrainment potential is governed by the size of a single SSE step (*rT*_*SSE*_*V*_*pl*_).

**Figure 5:**
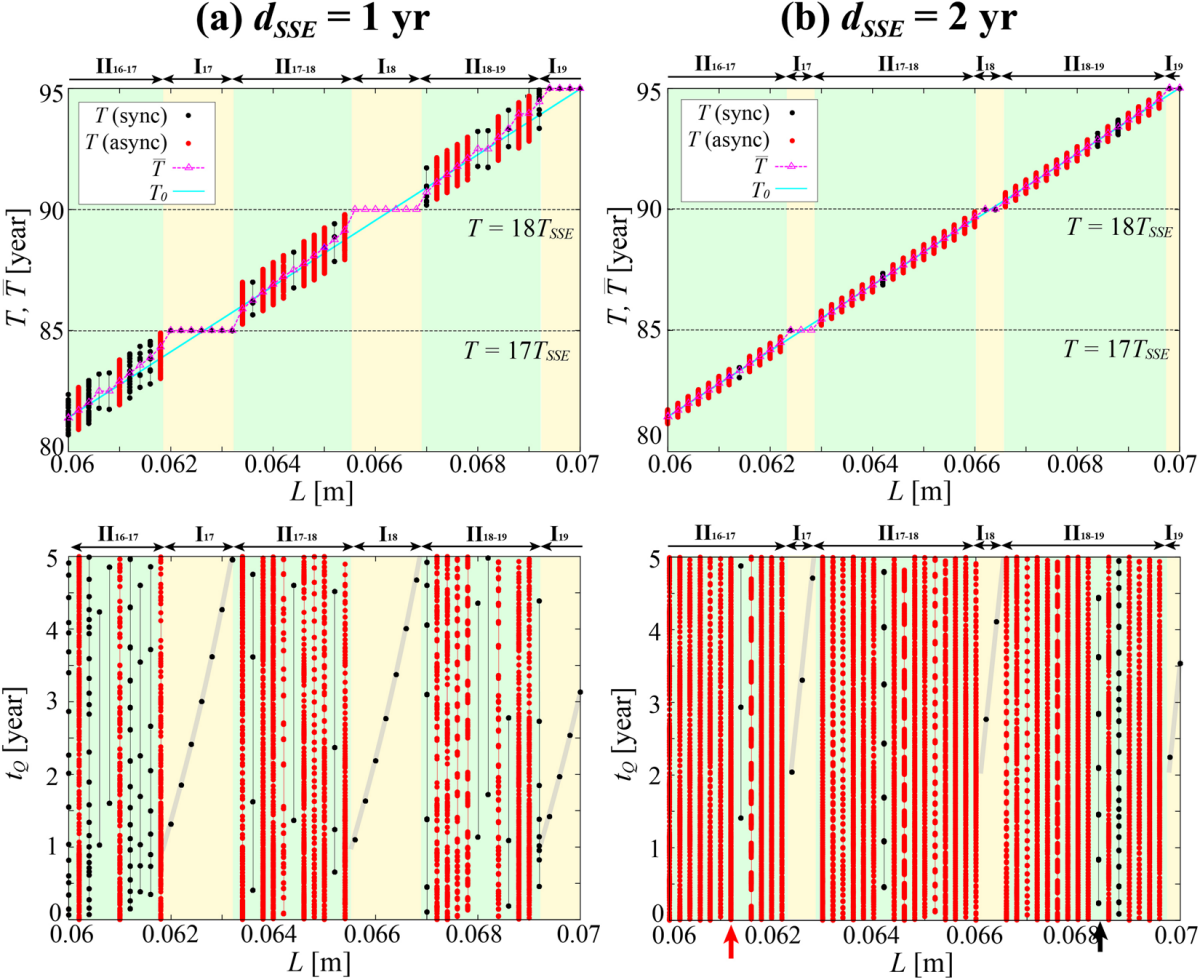
The *T*, $\bar{T}$ and *t*_*Q*_ [yr] in the case of *r* = 0.5 and *T*_*SSE*_ = 5 yr under (**a**) *d*_*SSE*_ = 1 yr and (**b**) *d*_*SSE*_ = 2 yr. See caption to Fig. 3 for details. The results (*T* in the top panels and *t*_*Q*_ in the bottom) for the synchronized and asynchronized cases are indicated by the black and red circles, respectively. In the bottom panel of (**b**), the black and red arrows indicate the examples of synchrony (*L* = 0.0684 m) and asynchrony (*L* = 0.0612 m) in regime (ii), respectively.

We further see that *S* does not depend on *T*_*SSE*_ because *T*_*SSE*_ dependences of numerator and denominator of Eq. (1) cancel out. A comparison between the cases of *T*_*SSE*_ = 1 yr (Fig. 4a) and 5 yr (Fig. 3b) shows, for example, that the size of a single SSE step is five times smaller in the former case than in the latter, so the width of an *L* interval under regime I_*m*_ is also five times smaller. By contrast, SSEs occur five times more often, which translates to five times more opportunities for entrainment into the SSE periodicity, so there are five times more I_*m*_ intervals per unit *L* interval. Combined, *S* remains unchanged overall, and understandably, does not depend on *T*_*SSE*_.

### Effect of *d*_*SSE*_

We are finally studying changes in the SSE duration *d*_*SSE*_. With *r* fixed at 0.5 and *T*_*SSE*_ at 5 yr, we studied the cases of *d*_*SSE*_ = 1 yr and 2 yr along with the earlier case of *d*_*SSE*_ = 0 (Fig. 3b). The results are shown in Fig. 5. The results for *d*_*SSE*_ = 1 month or so, which are not shown in the figure, were hardly distinguishable from the results for *d*_*SSE*_ = 0.

As readily seen from the existence of regime (i), synchronization occurred at least for certain *L* values even when *d*_*SSE*_ > 0 (Fig. 5). However, in regime (ii), synchronization occurs only for some *L* values (black circles). For example, synchronization with *n*_*EQ*_ = 7 is seen for *L* = 0.0684 m (black arrow) under *d*_*SSE*_ = 2 yr (Fig. 5b bottom). For the other *L* values, synchronization does not seem to occur (red circles). For example, for *L* = 0.0612 m under *d*_*SSE*_ = 2 yr (red arrow), no repeat pattern appeared throughout the entire experimented time (*t* = 10,000 yr–30,000 yr) worth 4,000 SSE cycles. We refer to this as asynchrony in the present study, although strictly speaking, we have not been able to make out for sure whether earthquakes and SSEs are still in sync in that case, only with immensely large *n*_*EQ*_ and *n*_*SSE*_ values beyond the tested number of SSE cycles, or they are really out of sync, with no periodic pattern whatsoever. For each *L* value with asynchrony, *t*_*Q*_ took a broad range of eligible values.

It was found that *S* = 0.60, 0.38 and 0.14, and *ΔT*_*max*_ = 1.51 yr (0.30*T*_*SSE*_), 0.97 yr (0.19*T*_*SSE*_) and 0.32 yr (0.06*T*_*SSE*_), when *d*_*SSE*_ = 0, 1 yr and 2 yr, respectively (Fig. 5 top). Equation (1) again holds true. The entrainment potential *ΔT*_*max*_ decreases with increasing SSE duration, which, combined with findings from the previous subsection, shows that *ΔT*_*max*_ = *f*(*d*_*SSE*_)*rT*_*SSE*_, where *f*(*d*_*SSE*_) is 0 < *f*(*d*_*SSE*_) < 1, a decreasing function of *d*_*SSE*_. Figure 5 top shows the variation of *T* at each *L* is bounded by 2*ΔT*_*max*_ constant throughout the experimented *L* range including both regime (i) and (ii), even when asynchrony occurs at some values of *L* in regime (ii). This suggests the concept of entrainment potential is valid even for the cases with asynchrony.

For regime (ii), we can define another measure of the prevalence of synchronization, *S*′; the proportion of the *L* values with synchrony among all the *L* values in the regime (ii). It was found *S*′ = 1.00, 0.45 and 0.09, when *d*_*SSE*_ = 0, 1 yr and 2 yr, respectively. Asynchrony becomes more common with increasing *d*_*SSE*_.

Comparing Figure 5b (*r* = 0.5, *T*_*SSE*_ = 5 yr and *d*_*SSE*_ = 2 yr) and Figure 3a (*r* = 0.1, *T*_*SSE*_ = 5 yr and *d*_*SSE*_ = 0), we notice the entrainment potential *ΔT*_*max*_ and *S* are about the same. Values of *L* with asynchrony, however, emerged only in the former case (*S*′ = 0.09), so it can be said the system is less prone to synchronization in the former case than in the latter. This suggests the presence of a certain effect of *d*_*SSE*_, on the prevalence of indirect synchronization in regime (ii).

In summary, the prevalence of direct synchronization (regime (i)), measured with *S*, is controlled by the entrainment potential *ΔT*_*max*_ = *f*(*d*_*SSE*_)*rT*_*SSE*_. Additionally, the prevalence of indirect synchronization within regime (ii), measured with *S*′, decreases with increasing *d*_*SSE*_.

## Discussion and Conclusion

We have used a spring-slider model to simulate the occurrence of earthquakes that are synchronized to the periodicity of loading by recurring SSEs. All seismic events in the experimented cases were found to be synchronized with SSEs when *d*_*SSE*_ = 0, and they were also found always to be in sync even when the single SSE size was small, such as when *r* = 0.1 and *T*_*SSE*_ = 5 yr. We could confirm synchronization down to *r* as small as 0.03 when *T*_*SSE*_ = 5 yr (figure not shown). When *d*_*SSE*_ > 0, by contrast, earthquakes were found to be out of sync with SSEs at certain *L* values. It is understandable that, the longer the SSE duration, the smaller the difference in the loading rate between when an SSE is going on and during the rest of the time, and hence the system becomes less prone to synchronization because an SSE becomes less of a special event or a clear time marker. When *r* = 0.5, *d*_*SSE*_ = 2 yr and *T*_*SSE*_ = 5 yr, for example, there is an ongoing SSE over 40% of all time, and the loading rate then is only 1.5 times the corresponding rate during the rest of the time, and asynchrony was found for the majority of the *L* values studied. Even in that case, however, synchronization was still found for some *L* values.

Two modes of synchronization were recognized when earthquakes were in sync with SSEs: regime (i) of direct entrainment into the periodicity of the SSEs, and regime (ii) of indirect entrainment, which involves the formation of a repeat pattern consisting of more than one seismic event. Periodic SSEs have the potential to shift an earthquake recurrence interval from the natural period *T*_0_ by up to the magnitude of their entrainment potential *ΔT*_*max*_. Whether the regime will be (i) or (ii) is determined by whether any *mT*_*SSE*_ values are available within that allowable range. Then *ΔT*_*max*_ controls the prevalence of direct synchronization, whereas the prevalence of indirect synchronization in regime (ii) is affected by *d*_*SSE*_.

The settings we have studied in the present article—(1) a smaller *r*, (2) a smaller *T*_*SSE*_ and (3) a larger *d*_*SSE*_—can all be regarded as a move toward the limit of the steady-loading model. The earthquake recurrence intervals *T* approached *T*_0_ under settings (1) and (3), and the system behavior approached that of the steady-loading model, wherein earthquake recurrence intervals vary continuously and linearly with *L*. No such approach, however, occurred under setting (2), because *S* does not depend directly on *T*_*SSE*_. Case (2) highlights a nonlinear nature of the synchronization system being studied here.

Our simulations over a wide range of settings have robustly exhibited synchronization of earthquakes to periodic SSEs. The appearance of synchronization means that the whole history of SSEs matters for the timing of individual earthquakes. Time delay of an earthquake from the most recent SSE is distributed broadly across the entire range of eligible values 0–*T*_*SSE*_. Thus, from the viewpoint of forecasting, the risk enhancement implied by the observation of an SSE is much less than that expected from the clock advance effect due to a single SSE imposed at an arbitrary timing^12^.

Future research could provide evidence of synchronization between earthquakes and SSEs in field data. The present article has shown that earthquakes may occur not necessarily at fixed recurrence intervals (regime (i)) but also in a complicated pattern of synchronization that involves more than one recurrence interval, so that may not give the impression at first glance that the seismic events are synchronized with SSEs (regime (ii)). As long as few SSEs and earthquakes have been observed, it remains difficult to discuss the relations between the SSEs and seismic events unless a clear spatiotemporal relationship is recognizable as in the Guerrero case mentioned in the introduction^10^. In the future, however, the complicated synchronization phenomena may be found in the modern observation data accumulated over a long time.

## Method

Under a pulling displacement *u*_*load*_ (Fig. 1b), the equilibrium of forces acting on the block (Fig. 1a) can be described quasi-dynamically with the following Eq. (2) of motion^20,21^ by using the spring constant *k*, the block displacement *u*, the block slip rate *V* and the friction *τ*:

2 $$\tau =k({u}_{load}-u)+\frac{GV}{2{V}_{s}},$$

where the S-wave velocity *V*_*s*_ = 3.27 km/s and the rigidity *G* = 30 GPa. We assume the friction *τ* follows a rate-and-state friction (RSF) law (Eq. (3)) and use the aging law (Eq. (4)) to serve as an evolution law for the strength parameter Φ^22^:

3 $$\tau ={\tau }_{\ast }+a{\sigma }_{n}\,\mathrm{ln}(V/{V}_{\ast })+{\rm{\Phi }},$$

4 $$\frac{d{\rm{\Phi }}}{dt}=\frac{b{\sigma }_{n}}{L/{V}_{\ast }}\exp (-\frac{{\rm{\Phi }}}{b{\sigma }_{n}})-\frac{b{\sigma }_{n}}{L/V},$$

where *a*, *b* and *L* are friction parameters, *σ*_*n*_ is the effective normal stress on the block’s sliding face, and V_*_ is an arbitrary reference velocity (we set V_*_ = *V*_*pl*_ in the present study). The τ_*_ refers to the frictional stress under the steady state with velocity V_*_.

By way of initial condition, we set *V* at 0.9*V*_*pl*_ and Φ at the corresponding steady-state value when *t* = 0. The first and second terms on the right-hand side of Eq. (4) represent the processes of time-dependent healing and slip weakening of the strength, respectively. The block motion in this system is generally takes the form of intermittent stick slip (earthquakes) when *a* < *b* and *k* < *k*_*critical*_ (≡ *σ*_*n*_(*b* − *a*)/*L*). We set *σ*_*n*_ at 100 MPa; *a* = 0.010 and *b* = 0.012; and *k* at 0.5*k*_*critical*_, because we are interested in earthquake occurrences. We use the Runge-Kutta method with variable time steps^23^ to solve the system of Eqs (2–4) to obtain time evolutions of the slip rate and the stress.

## Supplementary information

Supplementary

## Data Availability

No datasets were used in the current study.

## References

[1] Hirose H, Hirahara K, Kimata F, Fujii N, Miyazaki S (1999). A slow thrust slip event following the two 1996 Hyuganada Earthquakes beneath the Bungo Channel, southwest Japan. Geophys. Res. Lett..

[2] Dragert H, Wang K, James TS (2001). A silent slip event on the deeper Cascadia subduction interface. Science.

[3] Douglas A, Beavan J, Wallace L, Townend J (2005). Slow slip on the northern Hikurangi subduction interface, New Zealand. Geophys. Res. Lett..

[4] Schwartz SY, Rokosky JM (2007). Slow slip events and seismic tremor at circum-Pacific subduction zones. Reviews of Geophysics.

[5] Obara K, Kato A (2016). Connecting slow earthquakes to huge earthquakes. Science.

[6] Ozawa S, Murakami M, Kaidzu M, Hatanaka Y (2005). Transient crustal deformation in Tokai region, central Japan, until May 2004. Earth, Planets and Space,.

[7] Yamamoto E, Matsumura S, Ohkubo T (2005). A slow slip event in the Tokai area detected by tilt and seismic observation and its possible recurrence. Earth, Planets and Space.

[8] Kobayashi, A. & Yamamoto, T. Repetitive long-term slow slip events beneath the Bungo Channel, southwestern Japan, identified from leveling and sea level data from 1979 to 2008. *J*. *Geophys*. *Res*.*: Solid Earth*, **116**(B4) (2011).

[9] Ozawa S, Yarai H, Imakiire T, Tobita M (2013). Spatial and temporal evolution of the long-term slow slip in the Bungo Channel, Japan. Earth, Planets and Space.

[10] Radiguet M (2016). Triggering of the 2014 M_w_7.3 Papanoa earthquake by a slow slip event in Guerrero, Mexico. Nat. Geosci..

[11] Gomberg J, Beeler NM, Blanpied ML, Bodin P (1998). Earthquake triggering by transient and static deformations. J. Geophys. Res.: Solid Earth.

[12] Belardinelli, M. E., Bizzarri, A. & Cocco, M. Earthquake triggering by static and dynamic stress changes. *J*. *Geophys*. *Res*.*: Solid Earth*, **108**(B3) (2003).

[13] Kaneko, Y. & Lapusta, N. Variability of earthquake nucleation in continuum models of rate-and-state faults and implications for aftershock rates. *J*. *Geophys*. *Res: Solid Earth*, **113**(B12) (2008).

[14] Noda H, Nakatani M, Hori T (2013). A slower fault may produce a smaller preseismic moment rate: Non-1/tf acceleration of moment rate during nucleation and dependency on the background slip rate. Geophys. Res. Lett..

[15] Sugiura N, Hori T, Kawamura Y (2014). Synchronization of coupled stick-slip oscillators. Nonlin. Processes Geophys..

[16] Scholz CH (2010). Large earthquake triggering, clustering, and the synchronization of faults. Bulletin of the Seismological Society of America.

[17] Turcotte, D. L. *Fractals and chaos in geology and geophysics*. (Cambridge university press, Cambridge, 1997).

[18] Pikovsky, A., Rosenblum, M. & Kurths, J. *Synchronization: a universal concept in nonlinear sciences*, Vol. 12. (Cambridge university press, Cambridge, 2003).

[19] Bak P (1986). The Devil’s staircase. Phys. Today(United States).

[20] Rice JR (1993). Spatio-temporal complexity of slip on a fault. J. Geophys. Res..

[21] Segall P, Rice JR (1995). Dilatancy, compaction, and slip instability of a fluid-infiltrated fault. J. Geophys. Res..

[22] Nakatani M (2001). Conceptual and physical clarification of rate and state friction: Frictional sliding as a thermally activated rheology. J. Geophys. Res.: Solid Earth.

[23] Hairer, E., Nørsett, S. P. & Wanner, G. Solving Ordinary Differential Equations I - Nonstiff Problems, *2nd edn. Springer Series in Computational Mathematics*, Vol. 8. (Springer-Verlag, Heidelberg, 1993).

