# Supplementary material for: Synchronization of megathrust earthquakes to periodic slow slip events in a single-degree-of-freedom spring-slider model
Source: Sci Rep. 2019 Jun 4;9:8285. doi: 10.1038/s41598-019-44684-4 (PMC6547734; doi:10.1038/s41598-019-44684-4)
Supplement: Supplementary file 1 — Supplementary [file 41598_2019_44684_MOESM1_ESM.pdf]

Supplementary information

Synchronization of megathrust earthquakes to periodic slow slip events in a single-degree-of-freedom spring-slider model

Makiko Ohtani[1*], Nobuki Kame[2], and Masao Nakatani[2]

[1]Geological Survey of Japan, AIST, Central 7, 1-1-1 Higashi, Tsukuba, Ibaraki, JAPAN 305-8567

[2]Earthquake Research Institute, The University of Tokyo, 1-1-1 Yayoi, Bunkyo-ku, Tokyo, JAPAN 113-0032

[*]Corresponding author: ohtani.m@aist.go.jp

Intervals and timings of earthquakes at finely sampled L values

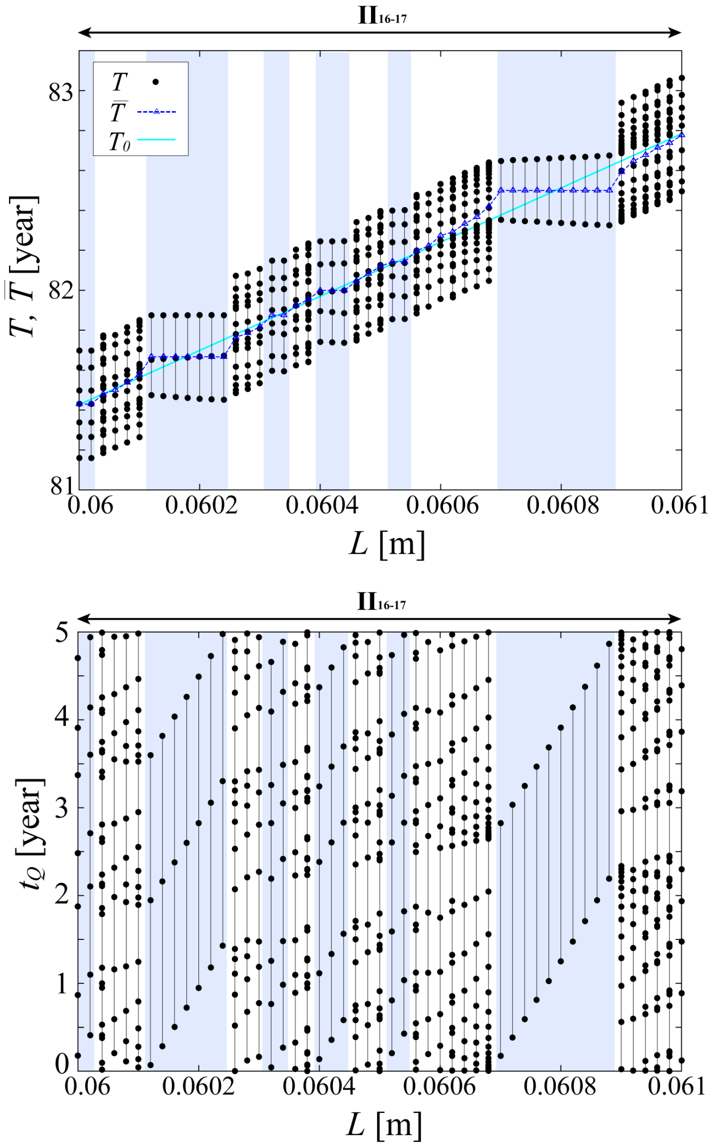

Figure S1. The T, $\bar{T}$ and t_Q [yr] in the case of r = 0.1, T_{SSE} = 10 yr and d_{SSE} = 0, for $0.06\ m \leq L \leq 0.061\ m$, which corresponds to a part of the interval $\mathrm{II}_{16\text{-}17}$. Same as a part of Fig. 3a except the finer sampling of L at intervals of 2×10^{-5} m. See caption to Fig. 3 for details. In the regimes colored blue, we can see step-like features in $\bar{T}$, which could not be recognized in Fig. 3a. In each blue regime, each of the multiple t_Q monotonically increases with increasing L, which is analogous to the trend of single-valued t_Q observed in regime (i).
